# The dynamic opponent relativity model: an integration and extension of capacity theory and existing theoretical perspectives on the neuropsychology of arousal and emotion

**DOI:** 10.1186/s40064-015-1120-6

**Published:** 2015-07-14

**Authors:** Clinton S Comer, Patti Kelly Harrison, David W Harrison

**Affiliations:** Department of Psychology, Behavioral Neuroscience Laboratory, Williams Hall, Virginia Polytechnic Institute and State University, Blacksburg, VA 24061-0436 USA

**Keywords:** Neuroscience, Brain asymmetry, Emotion, Arousal, Laterality, Cerebral balance theory, Opponent process theory, Anger, Sadness, Fear, Panic, Stress, Capacity theory, Neuropsychology

## Abstract

Arousal theory as discussed within the present paper refers to those mechanisms and neural systems involved in central nervous system activation and more specifically the systems involved in cortical activation. Historical progress in the evolution of arousal theory has led to a better understanding of the functional neural systems involved in arousal or activation processes and ultimately contributed much to our current theories of emotion. Despite evidence for the dynamic interplay between the left and right cerebral hemispheres, the concepts of cerebral balance and dynamic activation have been emphasized in the neuropsychological literature. A conceptual model is proposed herein that incorporates the unique contributions from multiple neuropsychological theories of arousal and emotion. It is argued that the cerebral hemispheres may play oppositional roles in emotion partially due to the differences in their functional specializations and in their persistence upon activation. In the presence of a threat or provocation, the right hemisphere may activate survival relevant responses partially derived from hemispheric specializations in arousal and emotional processing, including the mobilization of sympathetic drive to promote heightened blood pressure, heart rate, glucose mobilization and respiratory support necessary for the challenge. Oppositional processes and mechanisms are discussed, which may be relevant to the regulatory control over the survival response; however, the capacity of these systems is necessarily limited. A limited capacity mechanism is proposed, which is familiar within other physiological systems, including that providing for the prevention of muscular damage under exceptional demand. This capacity theory is proposed, wherein a link may be expected between exceptional stress within a neural system and damage to the neural system. These mechanisms are proposed to be relevant to emotion and emotional disorders. Discussion is provided on the possible role of currently applied therapeutic interventions for emotional disorders.

Over the last decade, research on emotion and arousal has shifted towards an integration of these constructs (Hagemann et al. 2003; Williamson and Harrison 2003; see Harrison 2015). This integration has presented the opportunity for controversy over the operational definitions of these constructs (Panksepp 2000), as well as controversy over certain assumptions involved in the study of emotion (Davidson 2003), both suggesting the need for more comprehensive models of emotion and arousal. The purpose of this review is not to pit any two perspectives against each other, rather the purpose of this paper is to trace through the logical development and progression of arousal/activation theory as it has been influenced through multiple eras of research regarding emotion.

The review begins by defining the functional cerebral systems approach and classic arousal theory. Prominent models of emotion that stemmed from arousal theory include the right hemisphere model, the valence and balance models, models of approach and avoidance, and dynamic activation models. Each of these models provides a unique contribution to the understanding of the functional cerebral systems of emotion and arousal. The integration that follows will present the utilities and limitations of each major theory and incorporate these elements into a proposed model of emotion and arousal. Moreover, this integration may provide for a better understanding of the processes involved in emotions such as hostility and fear, arousal related processes, and sympathetic processes including heart rate, blood pressure, and glucose metabolism.

## Classic arousal theory

The key to understanding how emotion and arousal are intertwined lies in functional cerebral systems theory. Within neuropsychology, functional cerebral systems theory, as proposed by Luria (1966), see (Harrison 2015), can be considered the equivalent of Einstein’s Theory of Relativity within the field of physics. This framework promotes the systematic exploration of functional interactions within the nervous system and has provided unsurpassed explanatory value and testability. On a behavioral level, research stemming from this theory has reported close associations between activation states of emotion and arousal. Likewise, neurobiological research has included the same cortical and subcortical structures when describing autonomic and emotional systems.

At the heart of Luria’s functional cerebral systems theory (Luria 1966) are Luria’s three principle functional units of the brain (Luria 1973). Luria proposed an organization of cerebral systems in which multiple units of the brain are connected through a functional hierarchy. The first functional unit is comprised of the brainstem reticular formation and is responsible for altering cortical tone and arousal. The second functional unit is comprised of the parietal, occipital, and temporal regions of the cortex, responsible for the reception, analysis, and comprehension of sensory information. The third functional unit is comprised of the anterior regions of the hemispheres (the frontal lobes) responsible for planning and sequencing, and regulatory control. Furthermore, multiple regions in different parts of the brain may be involved in similar tasks, and the activation of these areas will be similar across individuals.

Luria reported that “the nervous system as we know, always exhibits a certain tone of activity, and the maintenance of this tone is an essential feature of all biological activity. However, situations exist in which this ordinary tone is insufficient, and must be raised. These situations are the primary sources underlying activation” (Luria 1973, p. 52). Luria’s unique approach to explaining the functional interactions within the nervous system has been the framework of neuropsychological models of emotion and arousal. However, the application of this theoretical approach is incomplete. Understanding how the brain processes and acts on emotion, arousal, and various other processes is an active, cutting edge area of research at the present time.

Perhaps the most notable feature of functional cerebral systems theory is that it moved away from the notion of strict localization and toward a more systematic view of cerebral systems. Earlier efforts to explain the functional anatomy behind emotion had focused on the limbic system (Heilman et al. 1985; Papez 1937). Similarly, initial efforts to explain the biological proponents of arousal focused on the sympathetic and parasympathetic processes in the periphery. Such approaches were removed from Luria’s functional cerebral systems theory and the methods that followed from Luria’s theory shifted focus to the interconnectivity of the cerebral, brainstem, and cerebellar systems (Davidson 1993). This novel approach within neuropsychology, neurology, and psychophysiology led to prominent models of emotion and arousal.

A precursor to functional cerebral systems theory and likely the most fundamental theory underlying emotional processing within neuropsychology is Arousal Theory. Historically, research on arousal explored the relationships between the frontal lobes and the brainstem. The classic arousal literature focused on the diffuse projections of the mesencephalic reticular formation. Arousal theory was built upon research focused on the electrical stimulation of the reticular formation in the brainstem, which was found to result in the activation of higher level ipsilateral brain regions (Moruzzi and Magoun 1949). This revealed that the neural network that originates in the brainstem modulates the activation and arousal level of the ipsilateral hemisphere. The arousal systems of the reticular formation share connections with the anterior cerebral regions, which allow the frontal lobes to provide control over this system. Activation of this arousal system has been associated with cortical arousal (as evidenced by desynchronized EEG) (Moruzzi and Magoun 1949), behavioral arousal (e.g. wakefulness), and peripheral arousal through sympathetic measures (Lindsley 1960). Similarly, deactivation of this system has been associated with sleep, comatose states, and large amplitude, slow wave EEG.

Further research revealed that the activation of this system could also be regulated through manipulations in sensory input (Isaac 1960). In studies of nonhuman primates, ambient light and sound levels were found to directly alter arousal levels. Isaac and Devito (1958) found that reducing sensory input into the reticular formation improved the arousal irregularities that resulted from a prefrontal lobectomy. Shortly after, Isaac coined the term “arousal inhibitor”, describing the function of the frontal lobes.

Just as the reticular formation projects primarily to activate ipsilateral brain regions, the prefrontal regions exert their regulatory influence over the ipsilateral sensory and arousal regions, such that the right hemisphere regulatory capacity is strongest for the right sensory region (Knight et al. 1999). The frontal lobes have also been shown to modulate arousal components of sensory threshold (Harrell and Isaac 1969; Kallman and Isaac 1976) and modulate habituation to auditory and somatosensory stimulation (Butter 1964; Rule et al. 2002). Given the interface between the ipsilateral frontal lobes and reticular activating system, Luria (1966) noted that it was often difficult to distinguish frontal lobe dysfunction from brainstem and deep subcortical impairments. A major contribution of classic arousal theory and this early body of research on arousal is that it provided a basis for two separate arousal systems with the frontal lobes providing regulatory control. However, this line of research did not account for the asymmetry that exists between the arousal systems of the left and right hemispheres.

## The right hemisphere model

Arousal theory underwent a revolutionary change when Heilman and Van Den Abell (1980) posited that arousal should not be a symmetrical system, but rather that arousal should be lateralized differentially to the right brain. This followed Heilman and Van Den Abell’s (1979) right hemisphere model, which was originally derived from observing individuals with brain damage in either the right or left hemisphere (Heilman et al. 1975). Patients with right hemisphere lesions were found to have slower reaction times than patients with left hemisphere lesions. Consistent findings were demonstrated when normal subjects were shown to have faster reaction times to stimuli presented in the left hemispace than stimuli presented in the right hemispace (Heilman and Van Den Abell 1979). As a part of the right hemisphere model, Heilman incorporated the findings of previous research (Dimond and Beaumont 1973) that suggested that the left hemisphere performs vigilance tasks at a high level initially, but then declines, whereas the right hemisphere performs at an inferior but consistent level, thereby sustaining vigilance. Similarly, Konigsmark et al. (1958) and others (Moruzzi and Magoun 1949) had previously noted that the electrical stimulation of the right mesencephalic reticular formation resulted in activation that persisted longer than similar electrical stimulation of the left reticular formation.

The right hemisphere model posits that the right hemisphere plays a greater role in arousal consistent with its dominance in processing emotions regardless of valence (Heilman 1982). Sympathetic reactions to emotional events are also associated with right hemisphere activation, which is said to be the primary anatomical location for emotional comprehension and emotional expression (Heilman 1997). Such emotional dominance logically follows from the right hemisphere dominance for regulating bilateral cortical arousal levels (Howes and Boller 1975; Green and Hamilton 1976; Heilman et al. 1978; Heilman and Van Den Abell 1979; Heller 1993), as any intense emotion requires arousal or activation. Moreover, evidence exists for subliminal emotions (e.g., Gainotti 2012; Johnson 2005), where subcortical and brainstem mechanisms are involved in emotional processing. Regardless, of the level of analysis, the lateralization of emotion preferentially to the right cerebral hemisphere was presented numerous times within the literature by Heilman et al. (1985, 2003), Heilman and Bowers (1990), Heilman and Gilmore (1998) as well as others (Borod et al. 1983; Borod 1992; Bryden and Ley 1983; Buck 1984; Ross 1985; Tucker 1981a, b).

The notion of right hemispheric specialization for emotion can be traced back to Mills (1912a, b), who noted that patients with right hemisphere lesions displayed decreased emotional expression, and Babinski (1914), who reported that such patients were often indifferent. Also, patients with right temporoparietal lesions exhibited deficits in comprehending the emotional affect of speech (Heilman et al. 1975). While much of the early work leading up to the right hemisphere model focused primarily on the posterior regions of the cortex, especially the parietal lobes (Denny-Brown et al. 1952), researchers subsequently expanded its application into the frontal lobes (Heilman et al. 1993). This work lends itself to suggest that the right hemisphere maintains an excitatory role over the reticular activating system, and the left hemisphere possibly portrays an inhibitory role over the right hemisphere or the reticular activating system (Heilman 1997). Support for this notion has been found in patients with right-frontal lobe damage who have decreased regulatory control over emotions, (Heilman et al. 1993; Robinson et al. 1993; see Shenal et al. 2003, for a comprehensive review). Such right frontal lobe dysfunction is also associated with hyperarousal, as there is diminished regulatory control over the reticular formation via the descending projections as well as less regulatory control over the right posterior brain regions via the longitudinal tract (Shenal et al. 2003; Carmona et al. 2009; Foster et al. 2008).

Support for the right hemisphere model has grown to include right hemisphere dominance during emotional provocation (Borod et al. 1988; Tucker et al. 1977) and in the comprehension and expression of emotional prosodic speech (Borod et al. 1992, 1998, 2000, 2002; Bowers et al. 1987; Emerson et al. 1999; Heilman et al. 1975; Schmitt et al. 1997). Additionally, there is evidence for right hemisphere specialization in the perception of negative emotional faces (Herridge et al. 2004; Mandel et al. 1991; Wittling and Roschmann 1993) and in the expression of emotional facial gestures (Borod et al. 1997; Herridge et al. 2004; Rhodes et al. 2000).

Support also comes from findings that the left hemisphere attends to primarily right-sided stimuli, whereas the right hemisphere attends to stimuli within either hemispace (Heilman and Van Den Abell 1980). In the vast literature on neglect disorders, research findings are consistent that left hemispatial neglect is far more common than right hemispatial neglect, due to the attentional specialization of the right hemisphere (Heilman et al. 2003). Similarly, Borod (1992) suggests that these nonverbal, spatial, and integrative abilities of the right hemisphere give this hemisphere an advantage for processing emotions (see also Hagemann et al. 2005).

While studies of individuals with brain damage provide an understanding of the functional systems underlying emotion (Borod 1993; see Hagemann et al. 2005), research in non-brain damaged populations also yields information supporting the right hemisphere model. Consistent with studies of hemispatial neglect, high-hostile participants identified facial affect faster when faces were presented to the left visual field (right hemisphere) than to the right visual field (Harrison and Gorelczenko 1990). The literature on chimeric faces has provided robust evidence for the right hemisphere advantage for emotions (Levy et al. 1983; Bourne and McKay 2014). Likewise, in the auditory modality, a left ear advantage (right hemisphere) has been found for emotion identification (Bryden and MacRae 1989).

Electrophysiological and neuroimaging studies provide useful information regarding the right hemisphere specialization for emotion processing. Herridge et al. (2004) asked high-hostile participants to make angry facial expressions and found that the galvanic skin response (GSR) of these individuals was heightened and prolonged at the left hemibody (right hemisphere). Studies utilizing electroencephalography (EEG) have yielded greater relative right hemisphere activity during the processing of facial affect (Kestenbaum and Nelson 1992; Munte et al. 1998; Vanderploeg et al. 1987) and the processing of the emotional components of speech (Bostanov and Kotchoubey 2004; Everhart et al. 2003). More recent functional magnetic resonance imaging (fMRI) studies have found similar evidence for the right hemisphere’s involvement in the perception of facial emotion (Narumoto et al. 2001; Sato et al. 2004) and affective prosody (Buchanan et al. 2000; George et al. 1996; Imaizumi et al. 1997).

In a review of the literature, Silberman and Weingartner (1986) concluded that the largest amount of consistency supported the right hemisphere being dominant for emotion. There is abundant evidence supporting the notion that the perceptual and expressive processes regarding emotion, as well as the autonomic arousal processes, are asymmetrically represented in the cerebral hemispheres. Recent literature reviews of depression and other related studies continue to lend support to the model (Carmona et al. 2009; Demaree et al. 2005; Holland et al. 2014; Mollet and Harrison 2006; Kopp and Wessel 2008; Shenal et al. 2003; see also Harrison 2015).

## The valence and balance models

Differential hemispheric specialization for emotion dates back to Goldstein (1939), who reported “catastrophic reaction” in patients with left hemisphere lesions whereas patients with right hemisphere lesions were indifferent or euphoric. As support for the theories regarding arousal began to grow, theories pertaining to the left hemisphere’s involvement in emotion followed shortly after and led to a second major Hypothesis. In contrast to the right hemisphere model, the valence model posits that the right hemisphere is specialized for negative emotion and that the left hemisphere is specialized for positive emotion (Ehrlichman 1987; Silberman and Weingartner 1986; Borod 1992; Buck 1984; Heilman and Bowers 1990; Ross 1985). This model postulates that the right hemisphere is dominant in processing and expressing negative emotions and that the left hemisphere is dominant in processing and expressing positive emotions.

Before the valence model, evidence for right hemisphere involvement in negative emotion was plentiful. However evidence for left hemisphere involvement in positive emotion was more difficult to derive. For example, Dimond et al. (1976) presented motion pictures to either the left or right hemisphere through the use of special contact lenses which restricted vision to the half field. Films presented to the left half field (right hemisphere) were rated more negatively, which suggested that the right hemisphere is biased towards a negative evaluation of incoming stimuli. Ultimately, differential activation research was needed to begin supporting positive versus negative hemispheric contributions. Davidson et al. (1979) asked participants to continuously indicate their emotional responses to television programs. Left frontal activation was observed during positive affect and right frontal activity was observed during negative affect. Similarly, results were replicated when participants were asked to generate thoughts and feelings associated with positive or negative experiences. Consistency regarding these differential hemispheric specializations began to accumulate through studies of frontal activation (Ahern and Schwartz 1985; Jacobs and Snyder 1996; Tomarken et al. 1992).

Following the establishment of the valence model, theoretical bases followed from the findings of many researchers. These researchers sought possible explanations as to why the left and right hemispheres would be specialized for different emotions. One such explanation posited that negative emotions are linked with survival (Borod et al. 1986), whereas positive emotions are more linguistic and communicative (Borod et al. 1981). The notion of the left hemisphere pertaining to verbal communication and pleasantness carried the Valence Model into research on approach/withdrawal behaviors (Davidson 1984; Davidson et al. 1990; Fox 1991). However, the original predictions of the valence model were not lost. Tucker and Frederick (1989) expanded the valence model into the balance model of emotion.

While Heilman et al. (1993) had primarily looked at the effects of cerebral lesions, Tucker and Frederick (1989) discussed the effects of relative cerebral activation on emotions. The balance model provided a basis for deactivation of a particular hemisphere secondary to inhibitory influences of the homologous frontal lobe. Decreased activation of one hemisphere is posited to result in relative activation of the opposite hemisphere. Therefore, relative deactivation of the right cerebrum is said to result in increased relative activation of the left cerebrum which results in an increase of positive emotion. Similarly, relative deactivation of the left cerebrum is said to result in an increase of negative emotion. With this extension to the valence model, the balance model provides a framework for including deactivation of a functional cerebral system as an inherent part of emotion and arousal, thereby suggesting the need for a balance in these processes. Specifically, according the model, activation and deactivation occurs as a result of the system attempting to balance itself (Tucker 1981a, 1981b; see Mollet and Harrison 2006; Shenal et al. 2003 for reviews).

While the field was somewhat divided between the right hemisphere model and the valence/balance models, Silberman and Weingartner (1986) discussed the right hemisphere’s role in mediating negative emotions and the left hemisphere’s role in mediating positive emotions in a thorough review of the literature. The authors described that the right hemisphere would “retain dominance for controlling the balance between positive and negative affects, thereby controlling overall emotional tone” (p. 343). They suggested that there is not a true balance between the left and right hemispheres, likely due to the right hemisphere’s specialization for regulating cortical arousal, which is integral to emotion modulation (e.g., Papousek et al. 2009). Additionally, the review revealed that the field lacked the research needed to test the two hypotheses.

More recent studies have also provided support for the Balance Model. For example, massage therapy has been shown to decrease stress and increase left frontal activation on EEG (Diego et al. 2004), suggesting that reducing negative affect or increasing positive affect is related to left frontal activation. Additionally, participants with greater left frontal activation show an increased positive reaction to exercise when compared to those with greater right frontal activation (Petruzzello et al. 2001). Other studies of EEG have suggested that greater left frontal activation is associated with positive affect, whereas greater right frontal activation is associated with negative affect (Davidson 1995; Tomarken et al. 1990). Despite these findings, very few researchers have sought to address the assumptions of the Balance Model, especially when compared to the plethora of research specifically targeting the newer models of valence. Researchers pursuing subsequent models have provided a whole host of findings regarding relative activation, and the related homologous processes of the left and right hemispheres. These homologues will be revisited later throughout this paper. However, the result of this shift towards newer models of differential hemispheric activation is that the concept of “balance” has not been thoroughly assessed.

Also relevant to these discussions, the modified valence hypothesis (MVH) might be considered as a compromise between the right hemisphere model and the valence model. The MVH assumes a central role of inhibitory intra- and inter-hemispheric connections, which appears central to the discussions of our present models. Although it has not received much attention (Davidson 1984), more recently it has received partial support through an fMRI project (Killgore and Yurgelun-Todd 2007). In this project the models were tested, where the right posterior regions was found to activate to emotional face perception, irrespective of affective valence, albeit with greater activation to negative facial cues. Though less activated to emotional faces, the left posterior region appeared to recruit bilateral anterior brain regions in what the author’s describe as a valence-specific manner. The authors provide evidence for the concurrent operation of aspects of both of the models and they conclude that these two rival theories may not actually be in opposition, but that they may alternatively reflect different facets of a complex distributed emotion processing system.

Finally, we should mention the negative-only valence hypothesis (Najt et al. 2013), as well as the asymmetric inhibition model (Grimshaw and Carmel 2014). Beyond these newer developments, the reader might find the rather vast literature on split-brain patients to be helpful (e.g., Sperry 1961, 1982). Seminal studies on split-brain patients (e.g., LeDoux et al. 1977; Gazzaniga and LeDoux 1978; Sperry et al. 1979) and recent evidence might all be useful in understanding the present models (e.g., Prete et al. 2015).

## Activation theories

Following the valence and balance models, many researchers began reporting on the mechanisms that could be explained from these models through studies of brain activation. A new era of research emerged to test the behavioral correlates of activation of a particular hemisphere, and with advancements in methodological techniques, EEG usage became more prominent and fMRI usage emerged. Therefore the field adopted new terminology, where arousal and emotion were studied through activation states, which could be inferred from cortical desynchrony on the EEG and metabolic increments on the fMRI. Such studies led researchers to propose that the left anterior cerebrum processes positive emotional expression and that the right anterior cerebrum processes negative emotional expression (Davidson 1992a; Kinsbourne and Bemporad 1984). Positive emotional states have been associated with left hemisphere activity and negative emotional states have been associated with right hemisphere activity in a large collection of studies (Davidson and Fox 1982; Davidson and Henriques 2000; Davidson et al. 1979; Ekman and Davidson 1993; Ekman et al. 1990; Fox and Davidson 1988; Lee et al. 2004; Reuter-Lorenz and Davidson 1981; Schaffer et al. 1983; Sutton and Davidson 2000; Tomarken et al. 1992; Wheeler et al. 1993). For example, when asked to report emotional responses while watching a television program, EEG recordings demonstrated left hemisphere activity during positive emotional states, and right hemisphere activity during negative emotional states (Davidson et al. 1979). Similarly, infants have been observed to yield greater relative left frontal activity in response to viewing happy faces, and greater relative right frontal activity in response to viewing sad faces (Davidson and Fox 1982).

As theories of lateralized activation developed to explain emotion, further homologues between the left and right hemispheres were explored. During this era of research, additional models arose to account for the differences in relative activation that occur across broad categories of behavior. While a detailed explanation of all of these models is beyond the scope of the current review, the major contributions of such activation research will be noted. For example, dimensions such as motivation and behavioral approach/avoidance were a significant part of the activation literature, and led to the approach-withdrawal model. Davidson (1984) proposed that anterior left hemisphere activation was involved in social approach behaviors and that anterior right hemisphere activation was involved in social avoidance behaviors (see also Davidson et al. 1990; Davidson and Tomarken 1989; Fox and Davidson 1987, 1988; Carver and Harmon-Jones 2009). There is a great deal of overlap between emotional valence and social approach, as many negative emotions elicit withdrawal behavior and many positive emotions elicit approach behavior (Davidson 1995; However, see Carver and Harmon-Jones 2009). Therefore, Davidson and colleagues offered an explanation that the left and right frontal lobes were specialized for processing approach and withdrawal behaviors, respectively. Davidson et al. (1990) showed participants videos that were designed to elicit approach by inducing happiness or withdrawal by inducing disgust. When viewing the disgusting film, a significant shift toward greater relative right frontal activation was observed, as well as greater relative right anterior temporal arousal.

Consistent findings have been demonstrated for the expression of positive approach and negative withdrawal behaviors. Ekman and Davidson (1993) found that greater left hemisphere activation was associated more with the voluntary facial expression of a “Duchenne” or real smile, than the expression of a social or fake smile. These results suggest that the production of approach related expressions are also lateralized. More recent research supports the notion that withdrawal related facial expressions such as fear and disgust are associated with relatively less left frontal activation (Coan et al. 2001). Baseline frontal asymmetry has also been used as an indicator of approach and withdrawal tendencies. Fox et al. (1995) found that children who had a greater left frontal activation at baseline exhibited more social initiation and positive affect, whereas children with greater right frontal activation at baseline tended to remain more isolated. Resting frontal asymmetry has also been reported to be related to affective style in the adult population (Davidson 1992a, b, 1993, 1995).

Another important set of homologues between the left and right hemispheres are the differences in the types of information processing specialization and their processing styles. The left hemisphere is said to process information in a sequential, analytic manner, whereas the right hemisphere uses a simultaneous holistic style of processing (Bradshaw and Nettleton 1981).The left hemisphere is widely known for rapid, impersistent processing with a specialization for logical, linguistic speech, while the right hemisphere is widely known for slow, persistent processing with a specialization for spatial information such as faces and places. These differences in processing style further support approach and withdrawal models. For example, the fine motor control of the left frontal lobe may be useful in sequential social approach behavior, whereas the more global and automatic gross motor control of the right frontal lobe is useful in social withdrawal behaviors (Davidson 1984).

Along with the presentation of the approach/withdrawal model, the BIS/BAS model was proposed (Gray 1982). According to this model the behavioral activation system (BAS) activates behavior to conditioned stimuli to gain reinforcement or to avoid punishment, and the behavioral inhibition system (BIS) inhibits behavior to novel stimuli. Unlike the approach/withdrawal model, The BIS/BAS model places approach and active avoidance behaviors within the same system. For many researchers, this model provides a better explanation of the systems involved during frontal activation than the approach/withdrawal model (see Demaree et al. 2005, for a review). This stems from the issue that emotions of the same valence (positive or negative) do not always result in the same motivational behaviors (approach or withdrawal). For example, anger, when viewed as a tendency, may have an approach component, whereas fear is more likely to have a withdrawal component (Harmon-Jones et al. 2010; see also Carver and Harmon-Jones 2009). Research has supported that BAS scores are associated with left frontal activation (Harmon-Jones and Allen 1997; Sutton and Davidson 1997), whereas BIS scores are associated with right frontal activation (Sutton and Davidson 1997). However, inconsistent findings have led researchers to suggest that the two systems must be viewed on a continuum, where relative activation of one system and relative deactivation of the other system might be equally involved in emotion related behavior (Beauchaine 2001; Cox and Harrison 2008).

In addition to studies of approach and avoidance, researchers found hemispheric differences in autonomic arousal. Specifically, left hemisphere activation involves parasympathetic tone, whereas right hemisphere activation involves sympathetic tone (Carmona et al. 2009; Demaree and Harrison 1997; Herridge et al. 1997, 2004; Wittling 1990; Wittling and Genzel 1995; Wittling et al. 1998a, b; see Harrison 2015). As the right hemisphere is responsible for mediating arousal, vigilance, and outward-directed attention (Heilman 1995) and is activated by emotional situations of negative valence (Davidson 1995), it must also initiate and control the physiological stress mechanisms that occur with such events (Wittling 1997a). Wittling (1990) found that systolic and diastolic blood pressure increased significantly more when presenting a positively valenced emotional film to the right hemisphere than when presenting the same film to the left hemisphere. Similarly, Zamrini et al. (1990) examined epilepsy patients and found that heart rate increased following inactivation of the left hemisphere via intracartoid sodium amobarbital injection, whereas heart rate decreased following inactivation of the right hemisphere. Sensory stimulation of the right hemisphere has been found to increase cortisol secretion, whereas left hemisphere stimulation did not have an effect on cortisol levels (Wittling and Schweiger 1993).

## Active and dynamic processes

Shortly after the large body of research on the differential activation of hemispheric systems and frontal systems was established, another important concept was presented. Fox’s (1994) dynamic brain activity and emotion regulation model describes the anterior frontal regions as dynamic, active, and in opposition to each other. This new approach to hemispheric asymmetry emphasized the emotion and arousal systems as an active functional cerebral system, rather than a system to be activated. Fox described that changes in dysphoric affect could result from either right-sided excitation or left-sided inhibition. Additionally, he noted that one hemisphere may inhibit the affect-related behaviors of the other hemisphere. As a result, Fox concluded that notions of activity in one hemisphere versus activity in another hemisphere are inadequate ways to describe the dynamic changes within the brain; and when attempting to explain the role of hemispheric specialization, it is important to take into account this dynamic interaction.

The term “dynamic” had previously been used when describing the interactions between the left and right hemispheres (Tucker and Williamson 1984). However, the potential value of this construct did not appear to greatly influence the literature at the time. This is likely because Tucker and colleagues did not explain the role of dynamic interplay across the multiple methodological approaches to studying hemispheric specialization. Additionally, the role of dynamic interplay was not explained without making reference to the concept of “balance”. Specifically, Tucker and Williamson (1984) suggested that emotional processes dynamically regulate cognitive function through activation and arousal systems, yet balance was a fundamental assumption in their presentation of these systems. Davidson (1987) also reported on the dynamic interplay between the frontal regions through models of approach and avoidance behaviors. Despite previous failed attempts within the dense activation literature, Fox successfully integrated these findings and presented a model emphasizing the importance of this dynamic interplay between the two active and oppositional cerebral hemispheres.

Evidence for the dynamics between hemispheres can be traced back to early research on the emotional reaction that typically follows unilateral brain damage, as dynamics are represented through the ongoing communication and inhibitory processes of the two hemispheres. One such line of research comes from performing the WADA test on epilepsy patients. Sodium amytal was often injected into the carotid artery as an ipsilateral transient hemispheric anesthetic. Anesthesia of the right hemisphere has been characterized by a euphoric reaction, whereas anesthesia of the left hemisphere results in dysphoria (Lee et al. 1990). The emotional changes that were observed in patients following the WADA test have been interpreted to be the result of the release of one hemisphere from the inhibitory control of the other hemisphere. Similar conclusions have been drawn from studies of patients with unilateral brain damage. It has been argued that damage in one hemisphere releases the activity of the other hemisphere (Robinson et al. 1984). Sackeim et al. (1982) reported a greater probability of left hemisphere damage being associated with pathological crying, and right hemisphere damage being associated with pathological laughter.

In light of the ongoing communication between the left and right hemispheres, Fox (1994) argued that research on cerebral lateralization must take into consideration the dynamic interactions when explaining hemispheric specialization for emotion and emotion regulation. More recently, other researchers have acknowledged that the brain is an active and dynamic system. While describing a model of inhibition and sensitization, Thayer and Friedman (2002) argued that healthy biological systems do not function at an equilibrium or homeostatic state. Dynamic systems were said to consist of interconnections between subsystems, which allows them to work together in a coordinated fashion. Foster et al. (2008) discussed the dynamic system in the brain by stating that “activation or deactivation in one area of the brain may have specific, cascading interhemispheric and intrahemispheric effects on distal regions” (p. 2847). Similarly, Demaree et al. (2005) found evidence supporting a dynamic activation of the left and right hemispheres as a function of emotion. In a dichotic listening study, high hostile participants were found to have enhanced left ear detections, whereas low hostile participants were found to have enhanced right ear detections (Demaree and Harrison 1997) as a dynamic response to a painful event (cold pressor). EEG and fMRI research has also revealed the importance of dynamic interplay in behavioral control functions such as inhibition, task monitoring, and error detection (Garavan et al. 2002). Additionally, the ability of the frontal lobes to regulate information across domains such as emotion and cognition has been recognized as a dynamic filtering process (Rule et al. 2002; Shimamura 2000). Despite these more recent inclusions, the concept of a homeostatic brain is still prominent, and dynamic intercommunication has continued to be minimally acknowledged within the emotion and arousal literatures.

Another key concept of the dynamic brain activity and emotion regulation model (Fox 1994) is an appreciation of past findings that evidence the frontal regions as being regulatory over emotional behaviors. Fox posited that emotion regulation is a function of the dynamic interplay between the anterior regions of the left and right hemispheres. The model describes the posterior regions as responsible for evoking emotions, and the frontal regions as responsible for modulating emotions. This dual function is a product of the role that emotions play in our lives, as emotions need to be evoked and modulated. Therefore, the systems that evoke emotions are in opposition to the systems that modulate these emotions. For example, Tucker (1993) hypothesized that increased right frontal arousal represents increased inhibition of the right posterior region. Within the lesion literature, individuals with prefrontal lesions tend to be impulsive and poorly emotionally regulated (Kolb and Taylor 1990; Rolls et al. 1994; Tucker et al. 1995). Emotion regulation has also been linked to the developmental changes that take place in the frontal lobes during infancy (Dawson et al. 1992), and emotional control is reported to increase with age (Gross et al. 1997).

Additional models such as the circumplex model of emotion (Heller 1993) and the quadrant model of emotion (Shenal et al. 2003; Foster et al. 2008) have been developed to further explain the intrahemispheric and interhemispheric relationships between these dynamic oppositional systems. Such models propose that a dysfunction in any single hemispheric quadrant (left frontal, right frontal, left posterior or right posterior) will result in a change in all other quadrants. For example, frontal lobe dysfunction will result in decreased regulatory ability, leading to increased activation of the ipsilateral posterior region. This view of emotion regulation supports the functional cerebral systems approach, where the third functional unit (frontal lobes) is regulatory over the first (brainstem) and second (parietal, occipital, and temporal regions) functional units. It should be noted however, that the regulatory capacity of these systems controlling emotion is limited (e.g., Carmona et al. 2009; Holland et al. 2012, 2014; Klineburger and Harrison 2015; Mitchell and Harrison 2010; see Harrison 2015).

## Capacity model

We have proposed that frontal brain regions have limited capacity for regulatory control over oppositional neural systems. This defined capacity is viewed as dynamic and variable, whereas in the strictest sense the system might be viewed as working optimally in the presence of a single, low intensity emotional event or processing demand. In contrast, the system might degrade in capacity and even fail in regulatory control with multiple concurrent or sequential emotional events or subsequent to emotional events of heightened intensity or saliency. Therefore, frontal activation, whether it be through a shift in electrical activity (e.g., qEEG) or through increments in metabolic activity (e.g., fMRI or PET), is not unlimited. We have proposed that when capacity is exceeded, the system shuts down. This capacity model (e.g., Carmona et al. 2009; Holland et al. 2012, 2014; Klineburger and Harrison 2015; Mitchell and Harrison 2010, see Harrison 2015) may also be at the forefront of providing an explanation for long term neurocognitive damage resulting from the depletion within the system.

Within the neuropsychological framework of hostility, high hostile men have exhibited dysregulation of right cerebral systems, yielding an exaggerated sympathetic stress response through cardiovascular reactivity. For example, Foster et al. (2008) found that the right frontal lobe of high hostile individuals is unable to inhibit the right posterior region that regulates sympathetic activity when exposed to situational stressors. Specifically, when exposed to a cold pressor task involving the left hand (right hemisphere), heart rate and blood pressure increased in response to the stress. These individuals also evidenced decreased activity of the right frontal lobe and higher activity in the right posterior region on qEEG measures. Similarly, completion of nonverbal fluency tests, which is mediated by the right frontal lobe, has been shown to yield heightened systolic blood pressure in high hostile individuals (Williamson and Harrison 2003). When compared to low hostile men, high hostile men demonstrated interference effects on the task by committing more perseverative and organizational errors along with the increased systolic blood pressure. From a functional cerebral systems approach, this suggests that the frontal lobes have a “limited capacity” for regulating the posterior regions, while also mediating other frontal functions.

The cold pressor stimulus has been a useful stressor within the dual task paradigm. Demaree and Harrison (1997) administered an auditory dichotic listening test before and after the cold pressor. Following the stressor, high hostile men had increased blood pressure, heart rate, and correctly identified more single syllable word sounds at the left ear. The increase in right cerebral activation in high hostile men occurred with a corresponding increase in sympathetic tone. Increased arousal levels and heightened left ear advantage are indicative of increased right cerebral activation in high hostile men. Low hostiles showed lowered heart rate and blood pressure as well as a heightened right ear advantaged, demonstrating increased left cerebral activation to the stressor.

In the visual modality, hostile men and women were instructed to identify faces as angry, happy, or neutral, in either the right or left visual field (Harrison and Gorelczenko 1990). Hostile men and women were shown to have faster affect perception and negative perceptual bias, restricted to the left visual field. A replication of this experiment with the addition of the cold pressor found that hostile men demonstrated decreased accuracy in the recognition of emotional faces within the left visual field, while hostile men performed symmetrically across both visual fields.

More recent approaches within the dual task paradigm have used verbal and nonverbal fluency measures as frontal lobe stressors. The Controlled Oral Word Association Test (COWAT), a measure of verbal fluency or word generativity, has been shown to be sensitive to left frontal functioning (Benton and de Hamsher 1976), whereas the Ruff Figural Fluency Test (RFFT), a measure of nonverbal fluency or figural generativity, has been shown to be sensitive to right frontal functioning (Demakis and Harrison 1997; Foster et al. 2005). As noted above, Williamson and Harrison (2003) asked high and low hostile men to complete both of these lateralized frontal stressors, while evaluating cardiovascular reactivity. Results indicated that in hostile men, systolic blood pressure increased following the nonverbal stressor (RFFT), whereas systolic blood pressure decreased following the verbal stressor (COWAT). The authors found these results to be consistent with the capacity model (e.g., Carmona et al. 2009; Holland et al. 2012, 2014; Klineburger and Harrison 2015; Mitchell and Harrison 2010, see Harrison 2015), suggesting that the frontal regions were unable to regulate blood pressure with the concurrent demand of the stressor task.

To continue this line of research, Williamson et al. (Williamson, Harrison, Walters: The influence of lateralized stressors on cardiovascular regulation and dichotic listening in hostile men, in preparation) looked at the influence of hostility on cardiovascular regulation, verbal and nonverbal fluency, and dichotic listening. Blood pressure and heart rate measures were taken in high and low hostile men before and after verbal fluency tasks. It was predicted that high hostile men would exhibit cardiovascular activation subsequent to the nonverbal stressor but not the verbal stressor, indicating a diminished right frontal capacity. It was also predicted that high hostile men would exhibit a priming effect through a heightened left ear bias on the dichotic listening test following the nonverbal fluency task, but not the verbal fluency task. Consistent with predictions, high hostile men showed an increase in blood pressure when compared to baseline and low hostile men; they produced more perseverative errors on the task than low hostile men; and they displayed a priming effect at the left ear during the nonverbal fluency condition.

Similarly, high hostile men have been shown to have significantly higher blood glucose levels subsequent to the nonverbal stressor when compared to the verbal stressor (Walters and Harrison 2006b). This suggests that high hostile individuals are unable to concurrently regulate their glucose levels, while concurrently processing a right frontal lobe stressor. When considering these findings in light of the right hemisphere model, there should be a diminished capacity of the right frontal lobe to regulate the posterior systems of the right hemisphere (Walters and Harrison 2006b; Williamson and Harrison 2003; Williamson, Harrison, Walters: The influence of lateralized stressors on cardiovascular regulation and dichotic listening in hostile men, in preparation).

This capacity model is an extension of Kinsbourne’s functional cerebral space model (Kinsbourne 1980; Kinsbourne and Hicks 1978). Kinsbourne (1980) described that the cerebral systems share an organization network, and for this reason, dual tasks create interference effects, which are evidenced by a decline on concurrent task performance. Kinsbourne essentially extended the functional cerebral systems approach by addressing the notion of cerebral activation under challenging conditions. According to the model, the amount of interference on the concurrent performance of multiple tasks will depend on how related the tasks are and how close the involved cerebral networks are in physical space to each other. If the tasks are similar, and require networks that are in close proximity, performance will be enhanced through the ability to share networks. However if the tasks are dissimilar, but require the same networks, performance will be impaired due to the functional demands placed on these networks. As these functional demands increase, the cerebral capacity decreases and may be exceeded under extreme stress.

Interference is not the only assumption of the functional cerebral space model, as it also states that dual-task performance can be enhanced when tasks draw on resources from the same hemisphere (Hiscock and Kinsbourne 1977; Yazgan et al. 1995). When studying facial affect recognition, Root et al. (2006) presented emotional faces to both cerebral hemispheres, and had participants respond with either the left or the right hand. Results showed that reaction times were faster when the hemisphere processing the emotion and the response hand were congruent. Therefore, the right hand was found to be faster for responding to positive emotions, and the left hand was faster for responding to negative emotions.

As discussed earlier, the specific application of frontal lobe stress to high hostile individuals has continued to suggest a limited capacity of the frontal lobes through dysregulation of sympathetic arousal (Carmona et al. 2008; Mitchell and Harrison 2010; Walters and Harrison 2006a, b; Walters and Harrison: Frontal regulation of blood glucose levels as a function of hostility, submitted; Williamson and Harrison 2003). While limited capacity is a product of the dual task paradigm, when considering Tucker and Williamson’s (1984) balance model, limited capacity may also be a product of these functional cerebral systems being oppositional to each other. For example, the affective valence differences between the left and right hemispheres put these systems in opposition to each other.

## Opponent process theory

The association of oppositional processes in biological functioning is not without historical precedence. Heraclitus’ philosophy stated that all things are comprised of a pair of opposites (Bakalis 2005). This view was subsequently continued by Hegel and later by Marx (Stalin 1940). The concept was expanded into psychology, when Hurvich and Jameson (1957) proposed the opponent process theory to describe color vision. The most influential expansion of this theory followed shortly thereafter when the theory was used to explain concepts such as neural organization (Hurvich and Jameson 1974), emotion (Solomon and Corbit 1974), motivation (Solomon 1980), and addiction (Solomon and Corbit 1973). At its birth, the opponent process theory was a neuropsychological theory. It can be argued that opponent processes are prototypical representations of neurological processes. However, over recent decades the theory has been largely excluded or omitted from neuropsychological models.

While the opponent process theory was a psychological and neurological model proposed to account for a wide range of behaviors, emotion has been one of the most popular concepts explained by the theory. The theory asserts that processes (i.e. emotions) are paired, and when one emotion in a pair is experienced, the other is suppressed. In other words, if fear and pleasure are paired, when experiencing fear, pleasure is suppressed. These pairs are made up of the A-process or primary process, and the B-process or opponent process. The primary process and opponent process remain at a level of neutrality until the primary process is activated by some emotional event. In order to have evidence of an opponent process, something must be presented (i.e. emotional stimulus) and something must be taken away (i.e. emotional stimulus removed). The primary process responds to the demands confronting the organism such as the presence of the emotional event, yet the opponent process is slower to activate. This temporal delay in activation of the opponent process allows the emotion paired with the primary process to be experienced. If the emotional event is negative, then fear or anger might be experienced. To prevent the level of fear from becoming too great, the opponent process activates to suppress the primary process. At this point, the level of fear may diminish slightly, but does not return to baseline levels. According to the theory, a homeostasis is reached until the event changes or the process comes to an end.

The theory predicts a reaction in the opposite direction (pleasure) after the primary process (fear) has been reduced through habituation or other mechanisms. Once the emotional event ends, the primary process withdraws, yet the opponent process is slower to withdraw. This results in a residual feeling of pleasure shortly after experiencing fear. With repeated presentations, the opponent process is said to activate with heightened intensity and prolonged duration. Solomon and Corbit (1974) used this theory to account for the feelings of fear and pleasure that skydivers experienced; wherein veteran skydivers experienced less fear and more pleasure.

At the time of it’s development, the theory was also able to account for certain animal learning phenomena (Solomon and Corbit 1974), such as imprinting in ducklings (Starr 1978), Pavlovian conditioning in dogs (Overmier et al. 1979), and conditioning in rats (Maier et al. 1976; Rosellini and Lashley 1982) Another branch of the opponent process theory stemmed from this line of research and established itself in the classical conditioning literature (Schull 1979; Wagner 1981; Wagner and Brandon 1989). However this line of research is somewhat removed from the emotional predictions specified by Solomon, and is beyond the scope of this review.

Fundamental to opponent process theory is the concept of after-reactions to emotional events. This led early researchers to explore the hedonic contrast and affective dynamics involved in emotion. For example, Craig and Siegel (1979) administered a self report mood inventory to college students before and after a course final exam. The researchers hypothesized that any negative emotions prior to the exam would be followed by positive emotions following the exam. Mood ratings revealed that dysphoria decreased reliably and euphoria increased, which the authors interpreted to be in support of opponent process theory.

In another empirical test of oppositional emotions, Mauro (1988) hypnotized participants and led them to associate happiness and sadness with randomly assigned colored lights as stimuli. Additional colors were randomly assigned for after-sad and after-happy phases of the experiment. Physiological and self-report measures were taken before and during the presentation of the stimuli, while individuals were led to experience joy and sadness presented in a quasi-random order to control for expectancy effects. Heart rate and EMG (corrugator and zygomatic) data revealed that participants experienced sadness subsequent to joy, but did not experience joy subsequent to sadness. The authors concluded that emotions involving more intense physiological processes such as fear and joy are needed to observe the after-reaction, because the after-reaction is a product of the opponent process being unable to compensate for the sudden withdrawal of the primary process. In situations such as sadness, where physiological processes are less intense, the primary process decreases slowly, which might allow the opponent process to compensate, thus no after-reaction is observed. Given this interpretation, it logically follows that induced depression has also given researchers difficulty when attempting to observe after-reactions (Ranieri and Zeiss 1984).

Solomon described the sequence of emotions that he observed as “temporal dynamics”. As discussed previously, the concept of dynamic brain activity has remained underappreciated, and the same argument can be made for the concept of temporal dynamics. Despite the utility of the theory, especially in light of oppositional emotions, current inclusions of opponent process theory are almost nonexistent within the neuropsychological literature. This could be a result of the difficulties involved in designing a valid test of the theory.

For example, not all researchers have been able to support the opponent process theory. Sandvik et al. (1985) were unable to confirm the prediction that withdrawal responses become greater after habituation. Additionally, one finding of this study was a withdrawal response in the opposite direction than that which the theory predicts. Participants were required to listen to a story implementing imaginal exposure of good events or bad events across the domains of job situations, relationships, and other domains. Following these passages, participants rated their emotions using self report measures on which happy and unhappy were choices along a Likert scale. As previously mentioned, emotions that involve intense physiological processes are needed to observe after-reactions. Not only does “unhappy” lack the intensity required of this paradigm, but theoretically, oppositional emotions are not representing a continuous process, and should not be measured on a continuum. Furthermore, physiological measures are also essential to this type of research. In a review of the literature, Merckelbach et al. (1991) found that there was optimistic evidence for the theory. While empirical findings of studies were not unequivocally supportive of the theory, the methodological difficulties of this type of research were noted.

A more recent integration of opponent process theory tested the predictions of valence reversal following emotional stimuli using EEG. Kline et al. (2007) presented aversive images to participants and subsequently presented neutral images for a recovery phase. It was hypothesized that the affective contrast would be displayed in the asymmetrical activity patterns of the frontal lobes. Specifically, alpha activity, which is inversely related to the activation of the corresponding brain region, was compared across frontal regions. While aversive images did not produce a change in frontal asymmetry, participants with greater left frontal activity at baseline showed increased left frontal activity during the recovery phase. The authors concluded that participants with greater resting left frontal activation have better regulatory abilities over negative emotions, and an increase in left frontal activation following the aversive stimuli supported the concept of a lingering opponent process. Additionally, participants with greater relative right frontal activity at baseline show a relative inability to regulate negative emotional reactions, which is consistent with the literature on right frontal dysfunction.

Recent research of the capacity model yields promising evidence for the importance of opponent process theory. Solomon and Corbit (1978) also appreciated that the physiological resources involved in an opponent process system are not unlimited. According to opponent process theory, any prolonged or repeated departures from affective neutrality have a cost. To this effect, prolonged exercise or constant demand might lead to the exhaustion of a particular opponent process system. This implication is an extension of Selye’s stress theory (Selye 1950), which states that stress is broadly anything that sets into motion defense reactions, regardless of whether it is pleasurable or aversive.

## A proposed integration model for oppositional emotion and arousal systems

At the present, there appears to be a basis for looking at opponent process theory in light of the oppositional roles of the left and right hemispheres. We propose that the integration of opponent process theory and arousal/activation theory converges on four mechanisms. The first mechanism, arousal, is essential for the experience of any intense emotion. Therefore arousal systems must be incorporated into any functional cerebral systems model of emotion. The second mechanism is the right hemisphere’s specialization for threat detection and sympathetic response. The hemisphere that is specialized for these processes will be critical for evoking emotions and survival responses. The third mechanism lies in the differences in processing styles of the two hemispheres. As the right hemisphere activates through recruitment and persists longer than the left hemisphere, the emotions of the right hemisphere will be longer in duration than those of the left hemisphere (see Harrison 2015, Chapter 27). The fourth mechanism is the limited capacity of the systems involved in emotion and arousal processes, as oppositional systems may be depleted, resulting in deregulated affect.

While referencing a previous presentation, Tucker (1981a, b) described the two hemispheres in a manner consistent with opponent process theory, stating that “the two hemispheres seem to exist in some sort of reciprocally balancing, dialectical relationship with each hemisphere’s affective tendency opposing and complementing that of the other (p. 21)”. Tucker also noted that the right hemisphere was well connected with the subcortical arousal systems, whereas the left hemisphere appeared to exhibit inhibitory control over the right hemisphere. Despite this knowledge, Tucker’s balance model of emotion does not appreciate the contributions of opponent process theory. According to the balance model, relative activation in one hemisphere results in relative deactivation in the other hemisphere. This theoretical view of inhibitory processes arriving with activation in one hemisphere over activation processes within the homologous hemispheric regions may be criticized on a somewhat simplistic view of these neural systems. Should opponent process theory be accurate, then one contribution derived from the proposition of oppositional systems is that activation of the opponent system may be fully underway even during periods of maximal inhibition from the other system. Arousal theory provides a basis for this through bottom up and top down functional neural anatomy, first identified in the classic literature where the frontal lobe provides regulatory influences over the brainstem reticular activating system (Isaac 1960). This anatomy provides for a somewhat distinct and functionally separate arousal process within each cerebral hemisphere. Thus oppositional activation may arise somewhat independently within the left cerebral hemisphere despite the contralateral influences of an activated right cerebral hemisphere and vice versa. This view acknowledges that each cerebral hemisphere has somewhat distinct and unique arousal systems and provocative stimuli which may ultimately be oppositional to the processes of the other hemisphere.

Although the evidence for differential emotional processing is overwhelming, at this point, the largest consistency of evidence supports the right hemisphere model. Few researchers doubt the relationship between the right hemisphere and arousal/emotion. This fundamental inequity in the relationship between the left hemisphere and the right hemisphere contributions to these constructs requires alteration in the fundamental assumptions of the balance model. Specifically the inequity exists with an asymmetry in the right hemisphere, where the right hemisphere may be viewed as contributing arousal intensity and emotional specificity across all affective valences. In contrast, we propose a new model with a relative restriction and reduced weight on the “balance” scale with the left hemisphere contributions being of low arousal level, positive valence, and impersistent in temporal duration.

Also, there appears to be a basis for looking at the right hemisphere model again due to the current state of the balance model. Just as emotion, arousal, and activation were differentially lateralized to the right hemisphere, we should have a lateralized opponent process in the right hemisphere. When considering that the aforementioned arousal and emotion systems are dynamic and oppositional, where the right hemisphere is dominant overall, there will not be a true balance among these two brains that differ substantially in their processing styles. Just as spatial processing theory (Heilman and Valenstein 2003) posits that the right hemisphere is specialized in processing spatial information that is bilateral in origin, whereas the left hemisphere is restricted in processing minimal spatial information from the right hemispace. This same broad specialization of the right hemisphere for recognizing external threats in bilateral hemispace provides a fundamental compartmentalization for threat detection and comprehension of emotional events. We argue here that the same imbalance is necessary so that negative affective threat would persist indefinitely within the brain systems specialized for comprehending and responding to these potentially vital events, whereas the left brain contributes minimally to these processes, but instead to a rapid, sequential, linguistic, and positive demeanor of short temporal duration or brief in its persistence.

From the activation literature, we also have evidence that the left hemisphere is a rapid, sequential, and impersistent processor devoted to logical linguistic processing; in contrast, the right hemisphere is a slow, evolutionarily vital system, arranged to persist in its evaluation of potential threat or negative affective import. Thus, the two hemispheres can never be in balance under conditions of threat or coercion, and a relative dominance of the right hemisphere should exist. For survival, the negative emotions of the right hemisphere need to persist, whereas the positive emotions of the left hemisphere may be less persistent. Therefore, we propose a theoretical basis biased towards negative emotions, with dynamic activation of the left brain mediating the arousal and negative affect of the right brain. This is vital in situations of coercion or threat. We draw on this premise a fundamental assertion that the balance model is not truly balanced. One purpose of the present paper is to expose a potentially fundamental flaw in the balance model, where there cannot be balance between two oppositional states differing in speed and/or persistence of activation within these somewhat discrete functional cerebral systems.

To illustrate the difference in these processes, consider Aesop’s Fable, “The Tortoise and the Hare”. The tortoise is much like the right brain, and is working at full force much of the time, slow with its processes but persistent. The hare is intermittently working at full force, by being rapid with its energetic and sequential processing style, but does not persist for long periods of time. Looking across the entire race, we have a sense of balance between a slow, persistent racer, and a fast, impersistent racer, but at any given time, these racers are not truly in balance. This is similar to the two brains, where we propose an imbalance between these oppositional systems.

Here we propose a dynamic opponent relativity model that builds on over five decades of research incorporating the unique contributions from the aforementioned theories and models. This model asserts that each hemisphere has an independent arousal system, where the right hemisphere is specialized for negative emotion and threat detection. Additionally, the systems that involve positive and negative emotions are oppositional and active concurrently. These processes are dynamic, and have limited capacity. Taken in unison we have a model of emotion that describes the right hemisphere as dominant and for survival, and a left hemisphere that is specialized to minimize the effects of these emotions in a logical, sequential, analytic, and socially constructive way. When the right hemisphere is relatively activated, there should also be an undercurrent of the left hemisphere attempting to reduce the effects of the emotion.

**Figure 1:**
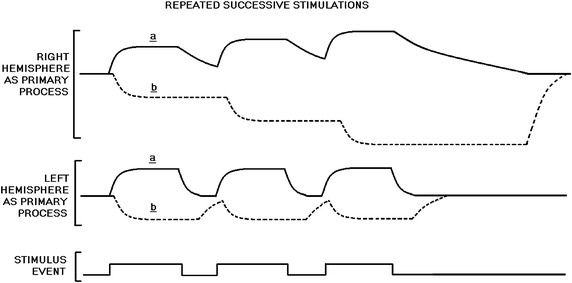
Differences in the persistence of activation of the right and left cerebral hemispheres. Emotional neutrality is more difficult to restore with repeated successive stimulations of the right hemisphere as opposed to repeated successive stimulations of the left hemisphere.

Staying true to opponent process theory requires the assumption that the visible effects of the opponent process will emerge following the resurgence of the primary process. Activation of a negative emotion such as anger may correspond with a buildup of the oppositional system, as predicted by opponent process theory. Following the withdrawal of the primary system there may be a biased post anger state, such as laughter or euphoria. When the effects of the primary system have been neutralized, the opponent process has been effective. While empirical support for after-reactions has been mixed, it should be taken into account that given the specialization of the right hemisphere, it will be a stronger oppositional process. As the right hemisphere is involved in intense negative emotions, and is designed to persist for survival, it will be harder to overtake in situations where it is the primary process. Also, in situations where the left hemisphere is the primary process, the right hemisphere will be better suited to reduce the effects of the primary process (see Figure 1).

This may explain why researchers have found that negative emotion follows positive emotion (Mauro 1988), but have not been unanimous in finding euphoric reactions after unpleasant events (Merckelbach et al. 1991).

As the dynamic opponent relativity model pertains to emotion and arousal, the implications to follow will mostly consider the right hemisphere to be the primary process. Given the specialization and persistence of the right hemisphere, it is better suited to restore emotional neutrality than the left hemisphere, which is specialized in less intense and less persistent positive emotions. Therefore, the right hemisphere offers potentially a superior opponent process, and is less likely to fail during periods of prolonged or intense happiness. While reverse configurations can be made, the model will be applied in situations involving negative emotion and elevated arousal with implied right hemispheric mechanisms. From a clinical perspective, the greater importance of this model lies in understanding the mechanisms involved during the exposure to aversive stimuli and negative emotions.

It should also be noted that the time frame over which these processes occur depends on the intensity and novelty of the experience. Opponent process theory predicts that the primary process does not withdraw until the emotional event ends (Solomon and Corbit 1973). Therefore a low intensity, short duration negative experience such as briefly viewing an angry face would result in a minimal sympathetic increase. These effects might be resolved by the opponent process in minutes or even seconds. However, more intense and prolonged provocation such as encountering an angry customer for 15 min would result in significant sympathetic increase. Such effects may take hours for the opponent process to completely resolve. In these examples, the sustained activation of sympathetic tone in response to possible threat can be explained by the recruitment and kindling phenomena of the right hemisphere (Stuss et al. 2001; Winston et al. 2002). Functional imaging studies of up-regulation have found that the right hemisphere recruits nearby areas such as the amygdale to activate, and an incremental, persistent “kindling effect” is observed (Hamann et al. 2002; Ochsner et al. 2004). This effect may also suggest that the right frontal lobes have less capacity than the left frontal lobes, as the actions of the right hemisphere’s arousal system are more slowly resolved.

In certain situations the opponent process may fail to resolve the primary process, as suggested by the limited capacity evidence. According to classic arousal theory, the performance of any function is best at some optimal state. This optimal state does not involve relative activation or arousal of the entire brain, but rather relative activation of a functional cerebral system. While these oppositional processes are dynamically responsive, active, intense and/or prolonged use may deplete the system, resulting in the failure of the opponent process. This means that one system, such as the right hemisphere, can be active, while another system is less active, which leads to a dominant negative expression, such as anger. However, once the opponent process becomes spent, there will be a release of the dominant emotion leading to rage for anger or terror for fear.

## Neurocognitive implications

We have proposed that these frontal systems have limited capacity for regulatory control over functional neural systems. Therefore, activation, whether it be through a shift in qEEG activity or through increments in metabolic activity, is not unlimited. We have proposed that when capacity is exceeded, the system shuts down. This capacity model (e.g., Carmona et al. 2009; Holland et al. 2012, 2014; Klineburger and Harrison 2015; Mitchell and Harrison 2010, see Harrison 2015) may also be at the forefront of providing an explanation for long term neurocognitive damage resulting from the depletion of a system. Further understanding of this limited capacity may be on the forefront of explaining the anatomical correlates of the brain that are affected by anger disorders, depressive disorders, and post traumatic stress disorder (PTSD).

With normal exposure to an emotional stressor, the opponent process is able to resolve the primary process. Furthermore, repeated normal exposures strengthen the opponent process and lead to an increased tolerance for stress. However, with intense or prolonged exposure, the opponent process may deplete its resources attempting to restore emotional neutrality. When this happens the primary system is no longer under the inhibitory influence of the opponent system, and the frontal lobe is left as the only regulatory system. While the frontal lobes are qualified for the regulation of emotion, by receiving sensory and motor input (Nauta 1971), as well as sharing connections with the amygdale (Pandya and Yeterian 1996), the literature on hostility has evidenced that the frontal lobes also have a limited capacity for stress. Along with the depletion of the opponent process, the regulatory frontal lobes of the primary process will also be depleted.

In the example of a single event trauma leading to PTSD in adults, a victim who is placed under intense and prolonged duress may experience startle, fear, feelings of helplessness, and even anger. These are all reactions and emotions associated with the primary process. If the event were intense enough to push the primary system too far, the opponent process would deplete its resources in an unsuccessful attempt to resolve the primary process. The unbridled sympathetic response and negative emotion that follows would essentially damage the emotion evoking and emotion regulating systems involved.

Research in PTSD has found evidence for long term neurocognitive damage. Specifically, exaggerated amygdale activation has been found in those with PTSD in response to traumatic reminders and threat predicting stimuli (Shin et al. 2004). As the amygdala is associated with fight or flight reactions, we would expect that it would activate in the presence of a threat. Similarly, Bremner et al. (1999) used PET and found deactivation in the medial prefrontal cortex in veterans with PTSD when presented with combat-related pictures and sounds. The amygdala’s shared connections with the ventromedial prefrontal cortex allow for regulatory control over the threat response, but with limited capacity. Also, combat veterans with PTSD have exhibited decreased right hippocampal volumes on MRI (Bremner et al. 1995) as well as decreased left and right hippocampal volumes (Gurvits et al. 1996) when compared to noncombat veterans or combat veterans without PTSD. The hippocampus is active in contextual memory, where the left hippocampus is specialized for encoding meaning and the right hippocampus is specialized for novelty detection. We propose that depleting the resources of the regulatory and excitatory systems in these situations is linked to such damage in the amygdale, prefrontal cortex, and hippocampus.

There is also evidence suggesting that when positive stimuli are presented immediately after a negative experience, cardiovascular recovery occurs faster than in the absence of positive stimuli (Fredrickson and Levenson 1998). Along this line, the current integration may provide the bases behind verbal cognitive therapies, where sequential, linguistic analysis of an emotion may activate the left hemisphere system which works in opposition to the negative emotional bias of the right brain. This inherently unbalanced–balance model should yield oscillating oppositional states due to the temporal differences and to the implied limitations in capacity, which should differ between the left and right brains. With this new model, we now have a system that is “balanced”, but biased towards survival.

In the absence of a threat, people spend a great deal of time in calm, quiescent states. Research on the “optimism bias” supports the notion that parasympathetic states and positive thoughts of the future are common and healthy (Sharot et al. 2007). Yet, in situations where there is an imbalance in the system where the right brain becomes ruminative and attentional processes are biased towards negative experiences, it may be very difficult for the left brain to reduce the right brain’s activation. However, through verbal therapies we can activate the logical, linguistic left brain. This may provide oppositional inhibition with a shift in focus towards positive experiences. For example, Fredrickson (2001) describes a broaden-and-build theory that suggests that positive emotions broaden one’s thought repertoire, undo lingering negative emotions, and build psychological resilience. The current model is consistent with such research and expands this view to posit that activation of the left hemisphere across a wide range of modalities will promote emotional well-being. In addition to positive emotion, sympathetic reduction, social approach, sequential processing, and linguistic speech are all behaviors that activate the left hemisphere. In addition to verbal processing, cognitive-behavioral therapies also implement techniques such as deep breathing, progressive muscle relaxation, and biofeedback. These exercises focus on reducing sympathetic processes such as heart rate, respiration rate, and galvanic skin response, suggesting that efforts to promote parasympathetic processes work in opposition to negative emotions.

There is reason to believe that regularly engaging in activities that strengthen the left hemisphere can act as a protective factor against negative emotion. Wittling (1997b) suggests that the left hemisphere is primarily responsible for modulating processes that help to maintain homeostasis, counteract environmental stress, and promote restorative processes. At baseline, relative right frontal activity has been viewed as a vulnerability marker for depression, whereas relative left frontal activity has been viewed as a protective factor (Henriques and Davidson 1990, 1991; Roemer et al. 1992; Gotlib et al. 1998; Nusslock et al. 2011; Stewart et al. 2011; Pössel et al. 2008; Shenal et al. 2003; see also Harrison 2015). Individuals with greater relative left frontal activity may be better able to regulate stress responses and emotional reactions, resulting in better overall resilience (Kline et al. 2007). Therefore, continued successful education or positive social encounters should provide the added value of activating the left hemisphere. While the left hemisphere is not specialized for survival and is incompetent for maintaining safety, increased left hemisphere activation may provide a better emotional disposition and decreases the risk of the right hemisphere taking over for chronic negative valence. As we further our understanding of the interdependence of the left and right brains, we propose that the field will need to shift into neurocognitive-behavioral therapies in order to benefit from integrated treatment approaches.

As previously noted, advances in neuroimaging techniques present new challenges for supporting the research that has been built upon over the last five decades. One such challenge is that newer language leaves a disconnection or sense of unrelatedness among those contributing to different research areas. Another challenge is that new lines of research leave older models with unequivocal evidence. This integration has shown that arousal theory has essentially evolved rather than having been left behind. For example, the classic arousal theorist would readily adopt the term activation through desynchrony on EEG measures, and the term metabolic rate in regard to fMRI measures. Common language is essential to being able to integrate the findings of researchers across multiple approaches.

## Conclusions

We have proposed that frontal brain regions have limited capacity for regulatory control over oppositional neural systems. Therefore, frontal activation, whether it be through a shift in electrical activity (e.g., qEEG) or through increments in metabolic activity (e.g., fMRI or PET), is not unlimited. We have proposed that when capacity is exceeded, the system shuts down. This Capacity Model (e.g., Carmona et al. 2009; Holland et al. 2012; Klineburger and Harrison 2015; Holland et al. 2014; Mitchell and Harrison 2010, see Harrison 2015) may also be at the forefront of providing an explanation for long term neurocognitive damage resulting from the depletion within the system. Further comprehension of these capacity limitations may be at the forefront of explaining the anatomical correlates of extreme emotional states, where anger, fear, or sad emotional expressions may become unbridled with the absence or the removal of regulatory control efforts. These discussions appear to be intimate to understanding anger disorders, depressive disorders, and post traumatic stress disorder (PTSD).

There is reason to believe that regularly engaging in activities that strengthen the left hemisphere can act as a protective factor against negative emotion. Wittling (1997b) suggests that the left hemisphere is primarily responsible for modulating processes that help to maintain homeostasis, counteract environmental stress, and promote restorative processes. At baseline, relative right frontal activity has been viewed as a vulnerability marker for depression, whereas relative left frontal activity has been viewed as a protective factor (Henriques and Davidson 1990, 1991; Roemer et al. 1992; Gotlib et al. 1998; see also Harrison 2015). Individuals with greater relative left frontal activity may be better able to regulate stress responses and emotional reactions, resulting in better overall resilience (Kline et al. 2007). Therefore, continued successful education or positive social encounters should provide the added value of activating the left hemisphere. While the left hemisphere is not specialized for survival and is incompetent for maintaining safety, increased left hemisphere activation may provide a better emotional disposition and decreases the risk of the right hemisphere taking over for chronic negative valence. As we further our understanding of the interdependence of the left and right brains, we propose that the field will need to shift into neurocognitive-behavioral therapies in order to benefit from integrated treatment approaches.
